# Capturing Genetic Variability and Identification of Promising Drought-Tolerant Lines in Exotic Landrace Derived Population Under Reproductive Drought Stress in Rice

**DOI:** 10.3389/fpls.2022.814774

**Published:** 2022-02-14

**Authors:** Challa Venkateshwarlu, Paresh Chandra Kole, Pronob J. Paul, Arun Kumar Singh, Vikas Kumar Singh, Arvind Kumar

**Affiliations:** ^1^International Rice Research Institute (IRRI), South Asia Hub, Patancheru, India; ^2^Institute of Agriculture, Viswa Bharati University, Sriniketan, India; ^3^International Crop Research Institute for the Semi-Arid Crops (ICRISAT), Patancheru, India

**Keywords:** drought-tolerant, abiotic stress, Chao Khaw, Kasturi, rainfed ecology, genetic variability

## Abstract

Drought is one of the most predominant abiotic stresses in this century, leading to a drastic reduction in the yield of rainfed rice ecosystems. Breeding of drought-resilient rice varieties is very much in demand for sustainable rice production in drought-prone rainfed ecology. An experiment was designed under irrigated non-stress and drought-stress situations involving an exotic drought-tolerant landrace (Chao Khaw) and a high-yielding aromatic rice cultivar (Kasturi), and an F_2:4_ derived population of 156 breeding lines was developed at IRRI South Asia Hub, Hyderabad. The objective of the study was to assess the genetic variability, drought tolerance behavior, and identify promising breeding lines for different rice ecologies and drought breeding programs. Restricted maximum likelihood (REML) analysis using the mixed model approach revealed a considerable genetic variation in the population for yield and yield contributing traits in non-stress and drought-stress conditions. We observed very high heritability for all the selected traits under stress 2015 WS (73.8% to 85.3%) and 2016 WS (72.4% to 93.5%) and non-stress 2015 WS (68.2% To 92.9%) and 2016 WS (61.4% to 92.6%) environments, indicating possible selection for grain yield under drought stress and non-stress with the same precision level. None of the secondary traits except harvest index and biomass included in our study showed a positive association with grain yield, indicating indirect selection’s ineffectiveness in improving yield under drought. A total of 48 promising breeding lines were found to have a better yield than donor Chao Khaw (up to 38% advantage) and popular drought-tolerant cultivars Shabhagidhan (up to 48% advantage) in stress conditions and recommended for rainfed upland ecology, 34 breeding lines under the well-watered condition suited for rainfed lowland ecology. Overall, the study found 21 common breeding lines that showed their superiority in non-stress and under drought stress situations, fitting best in rainfed lowland ecology with occasional drought occurrence. The large genetic variation found in this population can be exploited further to develop a few forward breeding high-yielding lines with better drought tolerance ability and used as drought donors in drought breeding programs.

## Introduction

Climate change is not a myth but a fact, which becomes a great threat to society due to its potential to intensify extreme events such as drought around the globe. Increased global temperatures and frequent changes in monsoon primarily cause the occasional droughts and floods, more common in many rice-growing areas in South and Southeast Asia.

Rice is the most widely consumed staple food as a cereal grain, is one of the most water-intensive crops, and grows about in the 25.12 million hectares area in irrigated conditions and 42.75 million hectares in lowland conditions in India. Moreover, rice consumes about 80% of irrigated freshwater resources worldwide (Wu et al., 2017). However, freshwater becomes increasingly scarce due to climate change and other factors such as rapid industrialization, urbanization, and groundwater mining (Bates et al., 2008; Xiong et al., 2010; Yan et al., 2015). According to a report, 15–20 million hectares of irrigated rice will face water scarcity by 2025 (Wu et al., 2017). Thus, it is a great challenge for the farmers to produce rice with limited water to meet the growing population’s food demand.

On the other hand, rainfed lowland ecology covers 33% of the rice area globally but yields only 19% of total global production (Rao et al., 2017). This comparatively low contribution toward total rice production of rainfed lowland ecology suffers from low productivity ranging from 1.5 to 2.5 tonnes (Rao et al., 2017). The unpredicted and frequent drought due to a disturbed monsoon or a long spell between two rainfall seasons causes the low productivity in this ecology. In Asia alone, about 34 million hectares of rainfed lowland and 8 million hectares of rainfed upland rice experience drought stress of varying intensities at different crop stages almost every year (Wopereis et al., 1996; Huke et al., 1997).

Many rice varieties grown in rainfed ecology are primarily developed for irrigated conditions and are preferred due to their high-yielding potential and better grain quality. These varieties are high yielding during the favorable season but perform poorly during the occurrence of occasional drought. Since the rainfed rice ecology is highly variable, breeding for drought-prone climate needs varieties that combine high-yielding potential with good drought tolerance (Zeigler and Puckridge, 1995). It is also imperative to breed better drought-equipped rice varieties with increased yield under drought conditions to encourage sustainable rice production in rainfed and irrigated ecosystems (Zeigler and Puckridge, 1995). A valuable and practical breeding approach for drought breeding and drought-tolerant rice varieties could lead to food security under growing food demands, limited resources, and unpredicted climatic variability.

The evolution of rice cultivars in drought-prone rainfed areas has allowed the development of a large number of drought-tolerant landraces which can withstand drought to many extents. Many of these landraces have also been known to possess deep roots up to 70 cm below the soil surface. Greater root length density at depth has also been reported in drought-tolerant landraces such as Dular, Azucena, and Rayada compared with high-yielding drought-susceptible varieties such as IR64 (Henry et al., 2011). However, most landraces have low-yielding potential, low tillering, tall plant height, and poor grain and eating quality. Despite being known to possess drought tolerance, very few of them have been systematically characterized for the trait. Previous decades have used many landraces to develop drought-tolerant cultivars and identify QTLs, for example, Basmati 370, PSBRc 80, Aus 257, Dhagad Deshi, Kali Aus Nagina 22, Aus Bak Tulsi, Dular (Wu et al., 2017).

Most traditional drought-tolerant donors are not used directly in breeding programs because of the linkage drag of several undesirable traits they possess. Hence, identifying and introgression of the genes or QTLs from them to existing elite varieties is the primary step for developing new generation drought-tolerant rice cultivars for rainfed lowland areas.

In the past, many drought-tolerant lines were developed by successful crosses between a drought-tolerant donor and a high-yielding drought-sensitive cultivar (Atlin, 2003). This study adopted the same method to attempt and produce drought-tolerant lines involving a drought-tolerant landrace collected from Laos and high-yielding aromatic rice with drought sensitivity. This population’s assessment would also help to study rice’s drought-tolerant behavior relating to exotic landrace as a donor. Besides, we were also interested in creating novel genetic variability for drought tolerance in rice to identify novel QTLs or genes for drought in further study.

## Materials and Methods

### Experimental Details

Experiments were conducted at the International Rice Research Institute (IRRI), South Asia Hub (IRRI-SAH), ICRISAT Campus, Hyderabad in 2015 WS (wet season/rainy season) and 2016 WS. The experimental plots are situated (78°16′ longitude, 17°32′ latitude, and 540m above sea level). The soil type of the conducted experimental plots was clay loam in texture. The non-stress experiments were sown during June and drought stress experiments were sown in July in 2015 WS and 2016 WS. To avoid the monsoon rain and maintain the drought cycle imposition, the sowings of the drought-stress experiment were delayed for one month from the non-stress experiment to get the severe stress cycles throughout the crop cycle. This drought imposition and drought screening method have been highly successful in the earlier study (Kumar et al., 2009).

### Plant Materials

Kasturi (RP 2144-108-5-3-2), long slender, improved aromatic variety for the irrigated lowland ecosystem variety, which has developed by selection from the cross-Basmati 370/CRR 88-17-1-5 at Indian Institute of Rice Research (IIRR), Rajendra Nagar, Hyderabad, was released for commercial cultivation in the year 1994. This variety is semidwarf in stature (95 cm), matures at 135 to 140 days, and yields about 30 to 35 quintals under the well-watered situation with an optimum nutrient application, which shows sensitivity during the reproductive stage drought situation. On the other hand, the donor parent, Chao Khaw, is a long bold Laos landrace, collected by the International Rice Research Institute germplasm collection and identified as a promising drought-tolerant genotype with blast-resistant having low grain quality traits, semitall in stature (110 cm) with medium duration (120 to 125 days) (IRRI unpublished data).

### Development of Population

The population was developed by crossing between Kasturi and Chao Khaw in the dry season (postrainy) of 2014, and 34 F_1_ seeds were generated. True single plants were harvested in 2014 WS, and 200 F_2_ individual plants were harvested in the dry season of 2015. Finally, in our study, 156 breeding lines in the F_2:3_ and F_2:4_ generations were included for screening for two main rice seasons in two conditions. IR 64, a high-yielding but drought-sensitive variety, and Sahabagidhan, a drought-tolerant variety, were planted along with the population in non-stress and reproductive stage drought-stress conditions.

### Phenotyping for Grain Yield Under Reproductive Stage Drought-Stress and Non-stress Conditions

**FIGURE 1:**
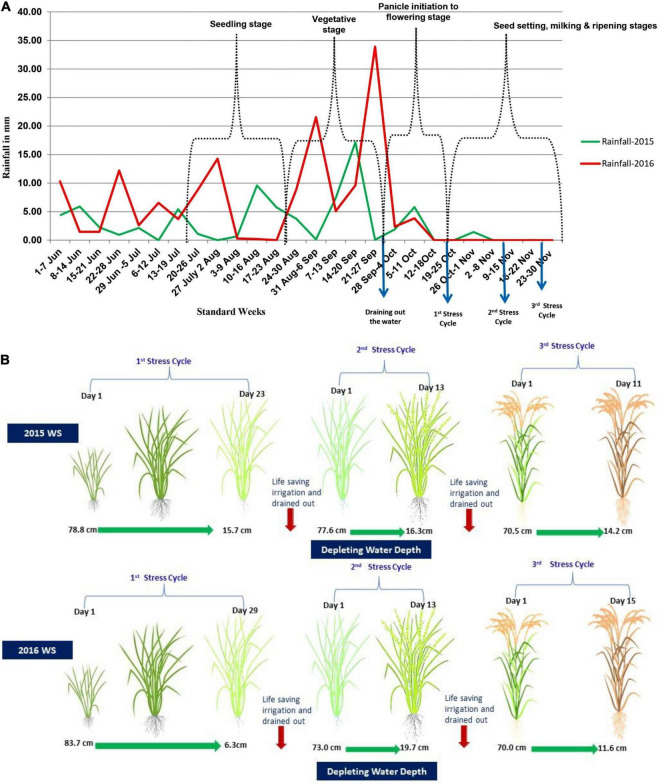
**(A)** Weekly rainfall pattern in the wet seasons of 2015 and 2016 at IRRI, Hyderabad, India. **(B)** showing water table content of the stress-imposed field in both seasons of 2015 WS and 2016 WS.

The phenotypic evaluation of the F_2:3_ and F_2:4_ population was conducted under well-watered and reproductive stage drought conditions. The field selected for the study was upland in topography with good drainage and loamy clay soil. Seeds were sown in a nursery bed and transplanted in the main field after 25 days of sowing under non-stress (well water condition) and reproductive stage drought-stress condition. Under non-stress and reproductive stage drought-stress conditions, each family was planted in two-row of 2 m with 20 cm × 15 cm spacing. The experiments were laid out with an augmented RCBD design with checks and parents repeated five times, and a standard package of practices was followed to raise the good crop. The total rainfall received during the crop cycle was 421.8 mm in 2015 WS and 834.6 mm in 2016 WS. The weekly rainfall pattern for 2015 WS and 2016 WS has been given in Figure 1A. To increase the chance that the drought experiment trials experienced drought-stress during the flowering stage, sowing time for drought trials was delayed about one month compared to the normal sowing time for this region. This practice was intended to postpone the flowering time to the end of the monsoon season. Non-stress condition trials were sown on June 23 and June 18, respectively, in 2015 WS and 2016 WS, whereas the sowing of drought experiment was on July 25, 2015 and July 20, 2016. Non-stress experiment was surface-flooded with irrigation to 1–5 cm depth throughout the crop growth till the ripening stage. Drought-stress experiment was maintained under the well-watered condition from transplanting until 45 days when drained out of the paddy field, and irrigation was withheld to impose the drought stress. The stress was continued until severe leaf rolling (LR) and tip burning were observed in at least 75% of the population lines, and water table depth depleted up to 75 to 100 cm below the soil surface for more than two weeks. After that, life-saving irrigation was provided through flash flooding, and water was drained after 24 h to impose a second cycle of drought stress. This kind of drought imposition cycle was repeated till harvesting (Figure 1B).

Water table depth was measured by installing a 1.1-m unplasticized polyvinyl chloride carbonate (UPVC) pipe in experimental fields at regular intervals. Depletion in the water table was measured through a meter scale daily after the onset of the stress. This irrigation regime resulted in stress levels that caused LR and tip burning on most entries at the end of each drying cycle. The repeated stress cycles ensured that all entries experienced stress during the sensitive stage of 15 days before and after flowering. On average, this protocol has been found to reduce yield by 50%–70% relative to fully irrigated controls (Venuprasad et al., 2007).

### Data Collection and Statistical Analysis

In all the trials, observation was recorded for days to 50% flowering (DTF), plant height (PHT), number of productive tillers (NPT), panicle length (PL), total number of grains per panicle (TGP), number of filled grains per panicle (TFGP), spikelet fertility percentage (SPF%), plot yield (PY), biomass (BM), and harvest index (HI). Data were recorded as per the standard evaluation system (IRRI SES, 2015).

The trait data in both stress and non-stress experiments were analyzed using restricted maximum likelihood (REML) methods for each season considering genotypes as a random effect and blocks as a fixed effect in the mixed-model procedure using GenStat for Windows 17th Edition, VSN International, Hemel Hempstead, United Kingdom^1^ (Patterson and Thompson, 1971). The variance components due to genotypes (σ^2^_*g*_) for each trait and their respective standard errors were determined. Best linear unbiased predictors (BLUPs) were estimated for each line in each experiment along with standard errors to test the significance of variance components. Further phenotypic correlations were estimated to determine trait associations in GenStat 17.

## Results

### Phenotypic Evaluation of Parental Lines and Population

Drought treatments in both years resulted in soil drying and subsequent yield reductions in parents and population compared with the well-watered control. The yield reduction in drought-tolerant parent Chao Khaw under drought-stress environments (DSE), DSE-2015, and DSE-2016 was 61.72% and 54.12%, respectively, whereas the reduction was much higher in drought-sensitive parent Kasturi in DSE-2015 (80.03%) and DSE-2016 (82.78%). The drought stress reduced 74.87% yield in the drought-tolerant cultivar Shabhagidhan in DSE-2015 and 70.35% in DSE-2016. Overall, the imposed stress caused mean reductions in yield of over 60% (60.24% in DSE-2015 and 64.86% in DSE-2016) in drought-stress trials than well-watered control. The population means for all the traits in the drought-stress environments were lower than the population means in the non-stress environment in both the years (Table 1).

**TABLE 1:** Genetic parameter estimation of various traits under non-stress and drought-stress conditions for Kasturi × Chao Khaw derived population.

Traits	Kasturi	Chao Khaw	Contrast analysis	Mean	SE	Min	Max	H^2^ (%)	GCV (%)	PCV (%)	GAM (%)

**Non-stress condition 2015**											
DTF (days)	104	91	13**	94	0.24	88	101	92.03	2.91	3.03	3.51
PHT (cm)	99.92	102.90	2.98**	88.45	0.57	75.60	108.19	92.95	7.75	7.78	6.03
NPT	10	7	3.00**	7	0.10	5	9	81.02	15.06	16.73	100.94
PL (cm)	25.70	22.06	3.64**	21.45	0.11	19.52	23.57	68.23	4.49	5.44	15.26
PY(g)	265.30	219.00	46.30**	263.31	3.59	201.90	344.30	83.03	14.16	15.54	0.21
BM (g)	511.00	472.40	38.60	587.89	11.82	370.00	1154.20	90.68	22.88	24.02	1.56
HI (%)	51.84	46.12	5.72**	45.95	0.52	28.30	51.10	78.02	11.00	12.45	12.31
TGP	176	157	19**	155	1.93	104	220	90.48	14.02	14.74	4.61
TFGP	160	142	18**	138	1.81	103	186	82.07	13.48	14.88	4.73
SPF%	91.33	90.03	1.30	88.82	0.43	75.54	95.19	81.69	4.95	5.48	4.43
GY (Kg/ha)	3316.00	2738.00	578.00**	3291.40	44.90	2524.00	4304.00	83.03	14.16	15.54	0.21

**Drought-stress condition 2015**											

DTF (days)	97	86	11**	90	0.25	86	94	73.81	2.56	2.98	2.9
PHT (cm)	84.74	67.55	17.19**	75.44	0.51	66.86	81.97	75.2	6.45	7.43	5.6
NPT	6	6	0.00	6	0.06	5	7	79.95	10.36	11.59	96.88
PL (cm)	21.06	18.3	2.76**	20.34	0.1	18.95	23.16	73.14	4.31	5.04	16.63
PY(g)	53	83.9	30.9**	104.69	3.01	55.8	143	82.72	29.77	32.73	0.75
BM (g)	184.9	179.7	5.2	270.09	7.91	120.3	497.2	85.16	31.16	33.76	3.77
HI (%)	27.53	47.07	19.54**	39.94	0.85	28.37	52.58	80.91	21.41	23.8	20.36
TGP	149	125	24**	120	1.74	86	154	77.85	14.23	16.13	5.38
TFGP	56	66	10**	63	0.92	42	85	85.35	15.47	16.75	11.41
SPF%	37.66	53.13	15.47**	52.83	0.34	48.73	57.1	84.93	6.77	7.34	8.97
GY (Kg/ha)	662	1048	386**	1308.61	37.7	698	1788	82.72	29.77	32.73	0.75

**Non-stress condition -2016**											

DTF (days)	108	91	18**	99	0.33	91	106	91.88	4.18	4.36	4.42
PHT (cm)	100.3	103.2	2.9**	109.89	0.66	89.5	126.9	92.62	8.95	9.3	6.77
NPT	10	6	4**	8	0.12	6	10	75.9	18.41	21.13	120.32
PL (cm)	26.44	25.29	1.15**	24.47	0.12	23.05	26.11	61.41	4.47	5.7	13.53
PY (g)	287.6	239.3	48.3**	362.62	4.25	206.6	495.7	83.55	13.86	15.16	0.17
BM (g)	745.3	537	208.3**	666.39	10.76	476.3	1172.8	81.25	36.9	40.94	3.61
HI (%)	39.84	45	5.16**	55.05	0.85	34.17	64.43	82.49	20.96	23.08	19.57
TGP	196	154	42**	165	2.29	127	246	88.56	20.11	21.37	6.69
TFGP	178	142	36**	142	2.05	102	209	83.72	29.47	32.21	13.44
SPF%	90.42	90.16	0.26	86.14	0.54	70.06	96.45	62.25	7.62	9.66	6.88
GY (Kg/ha)	3595	2991	604**	4017.78	53.1	2154	4950	83.55	13.86	15.16	0.17

**Drought-stress condition 2016**											

DTF (days)	100	84	16**	89	0.53	72	103	93.48	6.91	7.14	5.76
PHT (cm)	86.32	67.2	19.12**	86.28	0.66	71.48	105.99	91.6	8.79	9.19	6.63
NPT	5	6	1**	6	0.08	5	9	86.82	15.26	16.38	120.99
PL (cm)	23.68	24.25	0.57	22.28	0.13	19.91	24.14	72.4	5.41	6.36	16.87
PY (g)	49.5	109.8	60.3**	112.94	2.34	69.2	191.8	90.6	23.5	24.68	0.66
BM (g)	130.4	222.1	91.7**	299.29	9.54	161.7	589.2	89.07	35.45	37.56	3.76
HI (%)	40.57	50.45	9.88**	41.72	1.09	18.69	58.27	89.28	29.05	30.75	24.45
TGP	132	157	25**	126	1.35	105	168	85.15	11.38	12.33	4.9
TFGP	60	93	33**	73	0.89	54	93	85.36	13.02	14.1	9.07
SPF%	45.22	59.07	13.85**	57.96	0.4	48.47	63.29	82.81	7.18	7.89	8.27
GY (Kg/ha)	619	1372	753**	1411.68	29.3	865	2397	90.6	23.5	24.69	0.66

**DTF:** Days to 50% flowering; **PHT:** plant height; **NPT:** number of productive tillers; **PL:** panicle length; **PY:** plot yield; **BM:** biomass; **HI:** harvest index; **TGP:** total number of grains per panicle; **TFGP:** total number of filled grains per panicle; **SPF%:** spikelet fertility percentage; **GY:** yield in Kg/ha. **significant at P ≤ 0.01.

The mean values for DTF of the population in DSE-2015 and DSE-2016 were 4–10 days less than NSE-2015 and NSE-2016. As expected, the population mean for other traits, that is, PHT, NPT, PL, PY, BM, HI, TGP, TFGP, and SPF% in both stress environments, was lower than the non-stress environments. It is worth mentioning that the reduction from non-stress to stress environment was higher in TFGP than TGP, which indicates the role of fertility breakdown in the breeding lines under stress conditions.

**FIGURE 2:**
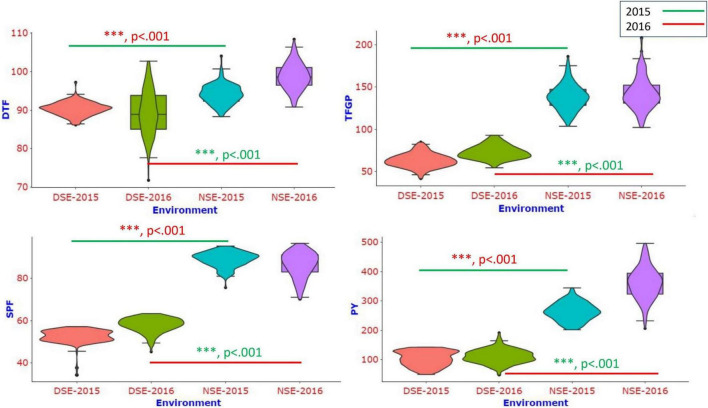
The violin plot shows the phenotypic distribution of DTF, TFGP, SPF% and PY traits in the population in different environments.

A single frame violin plot was drawn to visualize the phenotypic distribution of different traits across the environments for all the traits studied. The violin plots showed clear differentiation among stress and non-stress environments (Figure 2). The distribution shape of all the traits (skinny on each end and wide in the middle) indicates that the trait distribution is highly concentrated around the median.

Restricted maximum likelihood analysis for different components revealed that genotype variances were significant for all the traits studied based on the performance in individual drought-stress environments (2015 WS and 2016 WS) and the non-stress environments (2015 WS and 2016 WS). PY’s genotypic variance components (σ^2^g) were higher in NSE-2016 WS, followed by NSE-2015 WS than in DSE-2015 WS and DSE-2016 WS (Table 2). The σ^2^_*g*_ of SPF% for both the non-stress environments were almost the same and higher than their respective stress environments. As expected, TGP and TFGP had much higher σ^2^_*g*_ in non-stress conditions than drought-stress conditions. The phenology trait, DTF, showed maximum σ^2^_*g*_ components in DSE-2016 WS followed NSE-2016 WS, NSE-2015 WS, and DSE-2015 WS. The highest GCV and PCV values were recorded in BM with 31% and 33% in DSE-2015 and 35% and 37% in DSE-2016. This was followed by PY (29% & 32% and 23% & 24%) and HI (21% & 23% and 29% & 30%) in DSE-2015 and DSE-2016, respectively.

**TABLE 2:** Enotypic variance estimates and standard errors for all the traits measured in the developed population across two non-stress and drought-stress environments.

	Non-stress Environment 2015				Drought-stress Environment 2015				Non-stress Environment 2016				Drought-stress Environment 2016			
Traits	σ^2^_*g*_	SE	σ^2^_*e*_	SE	σ^2^_*g*_	SE	σ^2^_*e*_	SE	σ^2^_*g*_	SE	σ^2^_*e*_	SE	σ^2^_*g*_	SE	σ^2^_*e*_	SE
DTF	7.48**	1.14	1.59	0.53	5.33**	1.37	4.64	1.13	13.99**	2.08	3.03	0.96	38.09**	5.56	6.51	2.28
PHT	44.20**	6.58	8.21	2.84	23.66**	6.35	19.12	5.15	59.13**	8.57	11.54	3.68	57.58**	8.68	12.94	4.08
NPT	1.04**	0.23	0.60	0.17	0.36**	0.10	0.22	0.08	1.21**	0.35	0.94	0.28	0.83**	0.17	0.31	0.11
PL	0.93**	0.26	1.06	0.23	0.77**	0.26	0.69	0.22	0.99**	0.38	1.53	0.36	1.45**	0.45	1.36	0.38
PY	1388.20**	328.70	695.00	233.80	971.20**	218.50	497.20	155.20	1967.50**	445.00	948.90	311.50	704.10**	112.50	179.00	57.60
BM	17948**	2970.00	4517.00	1547.00	7081**	1452.00	3022.00	965.00	11960**	3133.00	6763.00	2322.00	11258**	2024.00	3384.00	1163.00
HI	25.67**	7.15	17.72	5.58	73.12**	18.99	42.26	14.14	76.41**	18.85	39.73	13.61	146.91**	26.97	43.21	15.52
TGP	473.90**	80.20	122.10	42.60	290.00**	75.30	202.20	58.70	642.1**	116.60	203.20	68.30	204.79**	43.34	87.49	29.01
TFGP	345.90**	85.80	185.20	62.40	95.20**	21.07	40.03	14.16	461.10**	100.70	219.60	69.80	89.86**	19.06	37.76	12.71
SPF%	19.36**	4.88	10.63	3.58	12.77**	2.10	5.55	1.31	19.45**	9.60	28.90	8.66	17.32**	3.97	8.81	2.83
GY(Kg/ha)	216900**	51354.00	108599.00	36531.00	151756**	34134.00	77692.00	24255.00	307425**	69530.00	148268.00	48665.00	110017**	17579	27977	9004

**DTF:** Days to 50% flowering; **PHT:** plant height; **NPT:** number of productive tillers; **PL:** panicle length; **PY:** plot yield; **BM:** biomass; **HI:** harvest index; **TGP:** total number of grains per panicle; **TFGP:** total number of filled grains per panicle; **SPF%:** spikelet fertility percentage; **GY:** yield in Kg/ha. **significant at P ≤ 0.01.

All the traits’ heritability estimates were very high in drought-stress environments and non-stress environments with a range of 73.14% to 85.35% in DSE-2015 and 72.4% to 93.48% in DSE-2016. All the traits in the non-stress trials, too, showed high heritability. The heritability of a few traits was greater in non-stress, and for some, it was greater in stress environments, which is consistent across the years. In general, there were more negligible differences in heritability between the stress environments of 2015 and 2016. Also, it should be noted that no significant differences in heritability were observed between stress and non-stress environments. In both the drought-stress conditions, only two traits, NPT (96.88% and 120.99% in DSE 2015 and DSE 2016, respectively) and HI (20.36% and 24.45% in DSE 2015 and DSE 2016, respectively), showed a very high genetic advance of mean (GAM).

**FIGURE 3:**
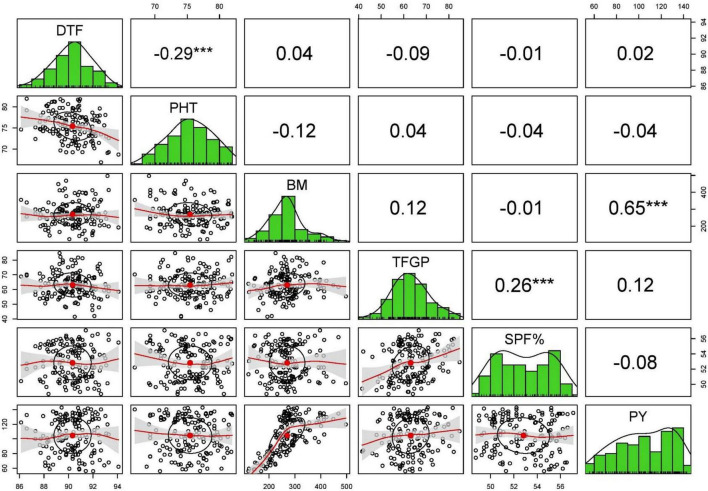
The scatterplot matrix shown here has histograms of the variables in the diagonal, and correlation coefficients in the upper part of the matrix under stress condition of 2015 WS.

**FIGURE 4:**
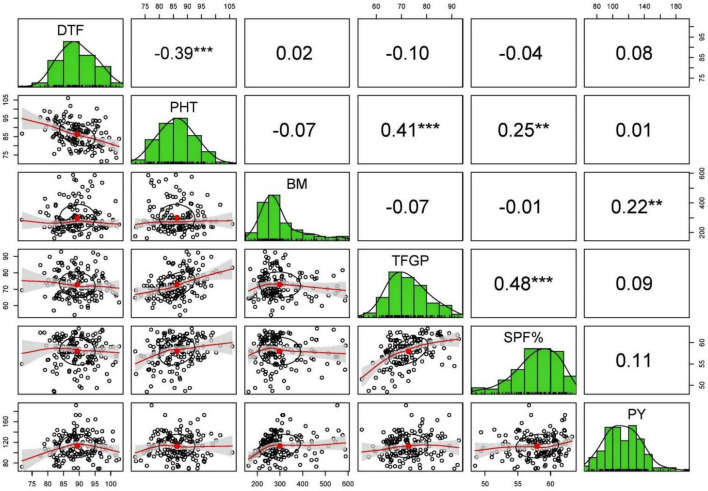
The scatterplot matrix shown here has histograms of the variables in the diagonal, and correlation coefficients in the upper part of the matrix under stress condition of 2016 WS.

The Pearson’s correlation analysis for different yield contributing traits under stress and non-stress conditions revealed that only BM and HI (0.22–0.65) were associated with grain yield in stress and non-stress environments (Supplementary Table 1). Days to flowering, plant height, number of panicles per tiller, panicle length, total number of grains per panicle, and total number of filled grains per panicle showed no correlation with grain yield in the non-stress environment and stress environments (Figures 3, 4).

### Identification of Promising Breeding Lines for Rainfed Lowland Ecology

In this experimental screening, we have found some transgressive segregants, which yielded much better than the popular mega variety, IR 64. These breeding lines may or may not have the drought-tolerant capacity but showed their yield superiority under well-watered conditions. A total of 34 breeding lines were found to have a significantly higher yield than the mega variety, IR 64. More than half of the breeding lines (21 lines) yielded more than 4,000 kg/ha. These breeding lines flowered earlier than the female parent Kasturi with 92 to 100 days falling in the medium maturity group and at par with IR 64 (Supplementary Table 2).

### Identification of Promising Breeding Lines for Rainfed Upland Ecology

Some drought-tolerant breeding lines for the areas in rainfed upland and highly drought-prone ecologies were identified from this study irrespective of their performance in the well-watered condition. We found 48 breeding lines with an average yield of 1,650 kg/ha and spans from 1,446 to 1,934 kg/ha. In contrast, the drought-tolerant popular variety Shabhagidhan and donor parent Chao Khaw yielded an average of 940 kg/ha and 1,210 kg/ha, respectively, over two years. The female parent Kasturi yielded just above half-ton per hectare. All the breeding lines under the drought stress condition were in the medium maturity group (86–96 days) with semidwarf plant type (73–91 cm). In contrast, the female parent Kasturi flowered in 104 days and the tolerant donor flowered in 91 days. The spikelet fertility of those breeding lines was 49%–58% under severe drought conditions (Supplementary Table 3).

### Promising Breeding Lines Suited for Rainfed Lowland Ecology With Occasional Drought Occurrence

This study identified 21 breeding lines common across the non-stress, and stress environments of 2015 WS and 2016 WS showed better yield performance than the tolerant check Shabhagidhan and tolerant donor Chao Khaw. These 21 breeding lines were selected from the 48 drought-tolerant promising breeding lines and 34 promising breeding lines in non-stress conditions. The top 14 breeding lines yielded more than 4,000 kg/ha (4,060 kg/ha to 4,363 kg/ha), and the average yield of the same 14 breeding lines, under drought stress, was 1,537 kg/ha (1,165 to 1,903 kg/ha) (Table 3).

**TABLE 3:** Promising high-yielding breeding lines identified under both non-stress and drought-stress conditions of 2015 WS and 2016 WS with better drought tolerance over the popular drought-tolerant check Shabhagidhan and drought-tolerant donor parent Chao Khaw.

		DTF		PHT		PY		BM		HI		TGP		TFGP		SPF%		GY (Kg/ha)	
Sl. No.	Promising Breeding lines	NS	DS	NS	DS	NS	DS	NS	DS	NS	DS	NS	DS	NS	DS	NS	DS	NS	DS
1	IR 128807-82-B	98	90	96.65	75.46	352.65	120.75	673.9	243.45	53.1	49.78	161	129	140	69	87.17	53.95	4364	1510
2	IR 128807-96-B	95	87	95.19	84.05	345.1	117.85	675.9	333.4	51.84	37.87	175	118	22	65	88.71	55.77	4289	1474
3	IR 128807-11-B	100	96	109.31	76.02	385.35	138.7	732.7	273.2	52.62	51.02	167	124	148	65	90.1	52.51	4269	1733
4	IR 128807-175-B	98	87	93.21	80.98	335.7	139.45	623.45	284.35	53.47	48.31	149	122	130	70	86.69	56.8	4217	1743
5	IR 128807-17-B	95	90	101.94	82.62	332.4	152.25	594.9	284.25	54.57	52.88	142	119	126	67	87.71	56.37	4200	1903
6	IR 128807-22-B	96	83	100.24	83.98	328.95	111.1	692.5	384.1	48.31	31.45	150	127	138	68	91.85	52.81	4143	1389
7	IR 128807-127-B	92	88	96.08	78.81	329.25	93.25	662.4	262.15	50.51	36.94	184	130	151	77	83.43	58.24	4143	1166
8	IR 128807-100-B	97	92	102.58	76.83	326.9	103.35	597.1	427.15	54.1	30.23	160	130	142	70	89.71	53.18	4136	1292
9	IR 128807-5-B	99	96	101.01	79.25	373.25	117.95	661.85	274.05	55.72	43.63	181	120	151	68	84.29	57.07	4113	1475
10	IR 128807-99-B	97	88	94.91	85.7	326.85	127.95	675.45	255.3	49.58	52.03	160	125	147	71	92.7	56.53	4111	1600
11	IR 128807-3-B	99	94	94.51	76	330.45	115.7	1124.6	364.1	35.64	39.55	158	119	142	63	89.72	53.06	4102	1446
12	IR 128807-12-B	97	90	101.95	79.55	324	149.5	637.5	304.05	50.85	49.15	175	121	138	69	79.04	56.61	4082	1869
13	IR 128807-46-B	96	94	102.7	85.18	327.75	115.65	579.75	249.75	55.38	47.42	181	122	160	67	89.77	54.99	4068	1446
14	IR 128807-155-B	96	90	102.72	80.09	321.4	118.4	582.75	284.5	54.25	41.95	139	103	121	57	85.24	55.81	4061	1480
15	IR 128807-147-B	96	94	92.29	79.82	317.6	97.2	648.15	219	49.41	45.25	160	126	143	72	88.25	57.05	3992	1216
16	IR 128807-73-B	99	94	98.79	83.04	316.6	119.7	630.5	245.85	50.37	49.62	202	118	166	66	85.23	56.13	3968	1496
17	IR 128807-16-B	97	89	102.31	85.2	327.65	128.75	665.35	265.95	51.05	49.66	146	131	126	74	85.1	56.53	3962	1610
18	IR 128807-38-B	95	91	99.97	83.31	324.55	123	595.95	259.25	54.5	49.09	164	132	139	73	84.38	55.37	3950	1538
19	IR 128807-163-B	96	90	99.51	81.24	348.85	136.85	621.7	430.05	54.34	34.07	141	122	117	66	80.75	54.13	3892	1711
20	IR 128807-39-B	95	89	96.93	78.75	316.95	135.9	601.45	277.65	53.17	49.25	163	137	138	80	84.6	57.75	3866	1699
21	IR 128807-95-B	95	90	101.95	80.14	309.8	139.3	628.6	286.4	50.1	48.7	159	122	144	71	91.27	58.31	3775	1742
	**Mean**	97	91	99.27	80.76	333.43	123.93	662.21	295.62	51.56	44.66	163	124	141	69	86.94	55.66	4081	1549
	**Min**	92	83	92.29	75.46	309.8	93.25	579.75	219	35.64	30.23	139	103	117	57	79.04	52.51	3775	1166
	**Max**	100	96	109.31	85.7	385.35	152.25	1124.6	430.05	55.72	52.88	202	137	166	80	92.7	58.31	4364	1903
	Chao Khaw (Donor parent)	91	85	103.05	67.375	229.15	96.85	504.70	200.9	45.56	48.76	156	141	141	79	90.10	56.1	2865	1210
	IR 64 (Check)	92	89	79.04	68.485	288.25	49.3	594.10	145.35	49.34	34.575	172	138	157	61	91.27	46.475	3603	617
	Kasturi (Recipient parent)	106	98	100.11	85.53	276.45	51.25	628.15	157.65	45.84	34.05	186	141	169	58	90.88	41.44	3456	641
	Shabhagidhan (Drought-tolerant check)	95	90	101.68	65.46	272.55	75.2	571.35	194.45	48.90	38.965	179	138	164	71	91.56	51.47	3407	940

***DTF:** Days to 50% flowering (days); **PHT:** plant height (cm); **NPT:** number of productive tillers; **PL:** panicle length (cm); **PY:** plot yield (g); **BM:** biomass (g); **HI:** harvest index (%); **TGP:** total number of grains per panicle; **TFGP:** total number of filled grains per panicle; **SPF%:** spikelet fertility percentage; **GY:** yield in Kg/h.*

Overall, all the 21 breeding lines yielded more than 3,500 kg/ha (3,775 kg/ha to 4,363 kg/ha) in non-stress conditions and more than 1,000 kg/ha (1,165 kg/ha to 1,903 kg/ha). In contrast, drought-tolerant Shabhagidhan had 3,407 kg/ha and 940 kg/ha in non-stress and drought stress conditions, respectively, whereas the donor parent Chao Khaw yielded 1,210 kg/ha in stress 2,864 kg/ha in a non-stress environment. Though Kasturi is a high-yielding variety, the yield was 640 kg/ha, which indicates its drought sensitivity under stress conditions. Interestingly, all the identified breeding lines flowered between 92 and 100 days in well-watered conditions and 83–96 days under stress conditions indicating they fall into medium-maturity groups. We also found that all the 21 breeding lines are semidwarf plant types (92–109 cm in non-stress and 75–86 cm in stress days). These breeding lines also have high tillering capacity with 6–8 productive tillers per hill.

## Discussion

Erratic rainfall distribution in rainfed areas and depleting the ground water level in irrigated areas threaten rice production globally and demand the urgent need to develop rice cultivars capable of producing more from diminishing water resources. The drought-tolerant rice varieties are believed to reduce rice production risk in rainfed areas, increase productivity in the changing climatic scenario, and alleviate poverty in rice-growing areas. Breeders need to adopt adapted solutions and strategies to respond to a different kind of drought situations based on the precise characteristics of different rice ecologies. For instance, the rice ecology of Eastern India receives sufficient rainfall, and so, rice is grown in rainfed conditions. However, due to erratic rain once in a few years, occasional drought leads to drastic yield reduction. Hence, rice drought breeding includes diverse approaches suitable for various rice ecologies and needs. For decades, traditional farmers have grown many landraces that may have low-yielding capacity but can mitigate the abiotic stress. Modern breeding strategies of elite x elite crosses for developing the high yielding ignore the need for landraces in the breeding programs. The underutilized landraces and their hidden treasures of tolerant genes/QTLs may help impart the maximum water-use efficiency in the elite modern breeding lines under alleviating the drought stress.

Though many studies have been conducted over the last two decades to develop drought-tolerant lines in rice, limited reports are in the public domain, where drought breeding programs involve exotic drought-tolerant landrace and popular aromatic cultivars. One such exotic landrace is Chao Khaw from Laos, which is considered an excellent drought-tolerant line with hidden drought-stress tolerant QTLs or genes. On the other hand, Kasturi, an aromatic rice variety, has high-yielding potential, excellent milling quality, blast resistance, and stem borer tolerance but is sensitive to water stress.

Drought screening, differentiating high-yielding and drought-sensitive lines from drought-tolerant ones and their yield gaps under non-stress and stress conditions, is considered adequate for selecting drought-tolerant higher-yielding lines. In this study, the drought stress experiments with imposed three cycles of stress and adjustment of sowing to avoid heavy rainfall, could accurately distinguish the drought-tolerant and drought-sensitive lines. The literature cites that if a lowland drought screening experiment reduces yield in the range of 30%, 31%–65%, and 65%–85%, then the drought should be considered mild, moderate, and severe types, respectively, and appropriate for drought tolerance breeding (Kumar et al., 2007). Yield reduction under drought stress is a common phenomenon reported in many studies earlier (Kamoshita et al., 2008; Ndjiondjop et al., 2010; Sandhu and Kumar, 2017; Bhattarai and Subudhi, 2018). The reduction of almost 80% yield in the sensitive parent Kasturi confirms the severity of drought of the two stress trials.

Along with the grain yield, other traits such as plant height, harvest index, spikelet fertility percentage, and the total number of filled grains showed reduced values under drought stress compared to the non-stress conditions. The reduction of spikelet fertility percentage under drought stress is considered crucial for yield reduction in rice under stress (Seyoum et al., 2012; Hasanuzzaman et al., 2014; Swamy et al., 2017). In this study, a reduction of about 30%–40% in spikelet fertility has been observed, subsequently, the yield reduction.

The mixed model REML analysis of variance revealed a significant variation among the breeding lines for all the traits across the environments. Furthermore, this confirms the fact that a good amount of segregation exists for the studied traits. Parents differed significantly for all the traits in both the drought-stress environments, which was also observed by previous studies (Venuprasad et al., 2007; Kumar et al., 2008). Parents’ significant difference in drought tolerance attributes, especially under drought-stress environments, validated the experimental conditions for evaluating drought tolerance breeding lines. The water depth level from the three stress cycles also confirmed the trials’ perfect drought-stress condition.

From the ranges of various traits across the environments and years, it is evident that most of the traits were higher in NSE-2016. The comparably high rainfall during 2016 non-stress conditions may be the reason for that. Under the drought stress conditions, except TFGP, the range of all traits was higher in DSE-2016 than in DSE-2015. The high groundwater level of the trial fields in all the three stress cycles in DSE-2016 was high compared to DSE-2015 which further confirms the large variability in DSE-2016 than DSE-2015. In short, the comparable favorable conditions of DSE-2016 were helpful in the expression of the breeding lines variably. It seems that the range of PHT and NPT was least affected across the trials except for DSE-2015. The relatively higher range of HI in drought-stress conditions indicates that there must be few breeding lines with a superior capacity of both sink and source and convert the photosynthates into usable economic yield.

All the breeding lines are grouped into the early to mid-early groups. Both the parents were from the early to medium category, and none of the parents was in the late category, which may be the reason for low variability in the population for flowering. Similarly, the low difference between parents for PH and PL may be responsible for the low variability of those traits. As expected, for PY, comparatively low variability was observed under non-stress conditions than the drought-stress conditions. High variability under the stress condition implicates stress-induced additional variability among the breeding lines through upregulation or downregulation of genes responsible for drought mitigation in plants. Like PY, HI also showed higher stress conditions than non-stress conditions reflecting the stress-induced variability in the population under drought-stress conditions.

The heritability estimates were found more or less similar in stress and non-stress environments and high for all the traits studied. No consistent reductions in heritability under severe drought stress were visible; in some cases, the heritability was higher in the stress environment. Heritability values of >70% for phenological traits and grain yield were also reported earlier (Vikram et al., 2011; Dixit et al., 2014a,b; Swamy et al., 2017; Solis et al., 2018). The relatively similar heritability estimates for grain yield under stress and non-stress situations indicate that selection for grain yield under water stress in rice is possible with the same level of precision as that achieved in non-stress (Kumar et al., 2008). This result confirms other reports that the heritability of grain yield under reproductive stage stress is comparable to that in non-stress trials (Lafitte and Courtois, 2000; Atlin and Lafitte, 2002; Babu et al., 2003; Atlin et al., 2004; Lanceras et al., 2004; Venuprasad et al., 2007; Kumar et al., 2008). This study also validates the hypothesis that yield evaluation under reproductive drought stress in rice can be conducted with a precision roughly equivalent to that obtained for non-stress trials and indicate that direct selection for grain yield under drought stress will result in gains if screening trials are well managed (Atlin and Lafitte, 2002).

The significant positive association observed between grain yield and harvest index under stress indicates that the yield differences we observed under drought stress were primarily due to the vast variation in plants’ capacity to maintain seed set under stress, apart from accumulating biomass. This result is consistent with previous studies, which reported that the reduction in yield when the drought stress is applied during flowering time is mainly due to spikelet sterility (Liu et al., 2006). As there was no correlation between days to flowering and grain yield in both non-stress and drought-stress conditions, it is evident that the drought tolerance shown by some breeding lines is due to the drought tolerance *per se* of those breeding lines than drought escape. There was no advantage of tall, semidwarf, or dwarf plants in influencing the yield under both non-stress and stress conditions as no association of plant height and grain yield was observed in both the conditions.

Similarly, in high yield, NPT and PL had no role, which indicate that breeding lines with more productive tillers and panicle length could have a more significant number of chaffy seeds or the weight of the seed was lower compared to other breeding lines with less productive tillers or panicle length but with high seed weight. As expected, in this studied population, TGP had no association with grain yield under both the conditions across the season, pointing out that the breeding lines behaved differently in producing filled grains. Many breeding lines might have many grains but many chaffy grains, whereas breeding lines may have few grains but more filled grains. This phenomenon is also reflected by spikelet fertility percentage as SPF% also showed no correlation with grain yield. There is no correlation between SPF% and grain yield which indicates that many low-yielding breeding lines are present in the population with high spikelet fertility. In other words, due to the smaller number of grains per panicle, these breeding lines could translocate the photosynthates to the majority of the grains. Except for biomass and harvest index, the absence of correlation with other traits with grain yield inferred that the breeding lines showed a very linear relationship with biomass and harvest index, but different breeding lines behaved differently with other traits. Hence, more biomass accumulation and plants’ capacity to maintain the good source-sink relationship under both conditions are beneficial to get a higher yield. No correlation between the number of filled grains and grain yield indicates the seed weight and the seed length, which may play an essential role in determining the grain yield rather than filled grains. The secondary traits included in this study were ineffective toward grain yield under stress and confirmed the earlier studies (Atlin and Lafitte, 2002; Kumar et al., 2008).

Two breeding lines yielded almost two times more than the tolerant check, Shabhagidhan, and 1.5 times the tolerant parent, Chao Khaw. This confirms that it is possible to select superior stress-tolerant breeding lines directly selecting grain yield under stress conditions from tolerant or sensitive crosses. These results have similarities with (Kumar et al., 2008), where they found breeding lines superior by 2–2.5 times than the tolerant parent. In the rainfed areas, farmers grow many traditional drought-tolerant but low-yielding cultivars that perform poorly if an occasional drought happens. The drought occurrence in Eastern India was almost two times in five years, whereas once in five years in northeast Thailand, an important rice ecology (Bhandari et al., 2007; Prapertchob et al., 2007). Hence, farmers need drought-tolerant varieties with high-yielding potential under favorable years but acceptable yield under moderate or severe drought conditions (Kumar et al., 2008). In our selection of drought-tolerant breeding lines in the populations, we observed transgressive segregants that yielded significantly higher than the parents under drought and well-watered conditions. These breeding lines were also found significantly better than the widespread drought-tolerant check Shabhagidhan. The promising breeding lines identified showed consistent performance across the years and hence are stable. Out of 21 breeding lines, 14 breeding lines yielded more than 4,000 kg/ha under the non-stress condition and yielded more than 1,474 kg/ha under severe drought stress. Under the same level of stress, the popular mega variety IR64 yielded only 616 kg/ha.

## Conclusion

In conclusion, this study revealed the availability of large genetic variation with high heritability in the studied population under both the drought-stress and non-stress conditions. The sufficient variation under drought stress confirms that the population was perfect for studying rice drought tolerance behavior. The imposed drought condition, which made the water depth level below the recommended level, provides the ideal condition for drought tolerance study. The studied population needs to be evaluated in multiseason and locations to know the significant genotype–environment interactions. The promising breeding lines identified suitable for different ecologies should extensively be evaluated under multilocation trials (MLTs) for immediate release as varieties or use as a donor in breeding programs.

## Data Availability Statement

The original contributions presented in the study are included in the article/Supplementary Material, further inquiries can be directed to the corresponding authors.

## Author Contributions

AK, PK, CV, and VS involved in conceptualization and methodology. CV contributed in field trial establishment and data collecting. CV, PP, and AS involved in statistical analysis. PP and VS contributed in writing original draft preparations. VS, AK, and PK involved in writing, reviewing, and editing. All authors have read and agreed to the published version of the manuscript.

## Conflict of Interest

The authors declare that the research was conducted in the absence of any commercial or financial relationships that could be construed as a potential conflict of interest.

## Publisher’s Note

All claims expressed in this article are solely those of the authors and do not necessarily represent those of their affiliated organizations, or those of the publisher, the editors and the reviewers. Any product that may be evaluated in this article, or claim that may be made by its manufacturer, is not guaranteed or endorsed by the publisher.

## Footnotes

1
www.GenStat.co.uk

## References

[B1] Atlin G. (2003). Improving drought tolerance by selecting for yield. Breeding Rice for Drought-prone Environments. *Int. Rice Res. Inst* 2003:14.

[B2] Atlin G. N. Lafitte H. R. (2002). “Marker-Assisted Breeding versus Direct Selection for Drought Tolerance in Rice,” in *Field Screening for Drought Tolerance in Crop Plants with Emphasis on Rice: Proceedings of an International Workshop on Field Screening for Drought Tolerance in Rice, ICRISAT, Patancheru, India, 11-14 Dec 2000*, (New York, NY: The Rockefeller Foundation), 71.

[B3] Atlin G. N. Lafitte R. Venuprasad R. Kumar R. Jongdee B. (2004). Heritability of rice yield under reproductive-stage drought stress, correlations across stress levels, and effects of selection: implications for drought tolerance breeding. *Resilient Crops Water Limit. Env.* 2004:85.

[B4] Babu R. C. Nguyen B. D. Chamarerk V. Shanmugasundaram P. Chezhian P. Jeyaprakash P. (2003). Genetic Analysis of Drought Resistance in Rice by Molecular Markers: Association between Secondary Traits and Field Performance. *Crop Sci.* 43 1457–1469. 10.2135/cropsci2003.1457 34798789

[B5] Bates B. C. Kundzewiez Z. W. Wu S. Palutikof J. P. (eds) (2008). *Climate Change and Water. Technical Paper of the Intergovernmental Panel on Climate Change.* Geneva: IPCC Secretariat, 210.

[B6] Bhandari H. Pandey S. Sharan R. Naik D. Hirway I. Taunk S. K. (2007). *Economic costs of drought and rice farmers’ drought-coping mechanisms in eastern India. Economic costs of drought and rice farmers’ coping mechanisms: a cross-country comparative analysis.* Los Banos: International Rice Research Institute (IRRI), 43–111.

[B7] Bhattarai U. Subudhi P. K. (2018). Identification of drought responsive QTLs during vegetative growth stage of rice using a saturated GBS-based SNP linkage map. *Euphytica* 214:38. 10.1007/s10681-018-2117-3

[B8] Dixit S. Singh A. Kumar A. (2014a). Rice Breeding for High Grain Yield under Drought: a Strategic Solution to a Complex Problem. *International Journal of Agronomy* 2014 1–15. 10.1155/2014/863683

[B9] Dixit S. Huang B. E. Sta Cruz Ma T. Maturan P. T. Ontoy J. C. E. (2014b). QTLs for Tolerance of Drought and Breeding for Tolerance of Abiotic and Biotic Stress: an Integrated Approach. *PLoS One* 9:e109574. 10.1371/journal.pone.0109574 25314587 PMC4196913

[B10] Hasanuzzaman M. Nahar K. Gill S. S. Fujita M. (2014). *Drought Stress Responses in Plants, Oxidative Stress, and Antioxidant Defense. Climate change and plant abiotic stress tolerance.* Weinheim: Wiley Blackwell, 209–250.

[B11] Henry A. Gowda V. R. P. Torres R. O. McNally K. L. Serraj R. (2011). Variation in root system architecture and drought response in rice (Oryza sativa): phenotyping of the OryzaSNP panel in rainfed lowland fields. *Field Crops Res.* 120 205–214. 10.1016/j.fcr.2010.10.003

[B12] Huke R. E. Huke E. H. (1997). *Rice Area by Type of Culture: South, Southeast, and East Asia.* Los Baños: IRRI.

[B13] IRRI SES (2015). *Standard Evaluation System for Rice (SES). IRRI SES-2015.* Available online at: http://www.knowledgebank.irri.org/images/docs/rice-standard-evaluation-system.pdf (Accessed on 11 Oct, 2021)

[B14] Kamoshita A. Babu R. C. Boopathi N. M. Fukai S. (2008). Phenotypic and genotypic analysis of drought-resistance traits for development of rice cultivars adapted to rainfed environments. *Field Crops Res.* 109 1–23. 10.1016/j.fcr.2008.06.010

[B15] Kumar A. Bernier J. Verulkar S. Lafitte H. R. Atlin G. N. (2008). Breeding for drought tolerance: direct selection for yield, response to selection and use of drought-tolerant donors in upland and lowland-adapted populations. *Field Crops Res.* 107 221–231. 10.1016/j.fcr.2008.02.007

[B16] Kumar A. Verulkar S. Dixit S. Chauhan B. Bernier J. Venuprasad R. (2009). Yield and yield-attributing traits of rice (Oryza sativa L.) under lowland drought and suitability of early vigor as a selection criterion. *Field Crops Res.* 114 99–107. 10.1016/j.fcr.2009.07.010

[B17] Kumar R. Venuprasad R. Atlin G. N. (2007). Genetic analysis of rainfed lowland rice drought tolerance under naturally-occurring stress in eastern India: heritability and QTL effects. *Field Crops Res.* 103 42–52. 10.1016/j.fcr.2007.04.013

[B18] Lafitte R. Courtois B. (2000). Genetic variation in performance under reproductive-stage water deficit in a doubled haploid rice population in upland fields. *CIMMYT* 2000:97.

[B19] Lanceras J. C. Pantuwan G. Jongdee B. Toojinda T. (2004). Quantitative Trait Loci Associated with Drought Tolerance at Reproductive Stage in Rice. *Plant Physiol.* 135 384–399. 10.1104/pp.103.035527 15122029 PMC429392

[B20] Liu H. Mei H. Yu X. Zou G. Liu G. Luo L. (2006). Towards improving the drought tolerance of rice in China. *Plant Genet. Resour.* 4 47–53. 10.1079/PGR2006111

[B21] Ndjiondjop M. N. Manneh B. Cissoko M. Drame N. K. Kakai R. G. Bocco R. (2010). Drought resistance in an interspecific backcross population of rice (Oryza spp.) derived from the cross WAB56-104 (O. sativa)×CG14 (O. glaberrima). *Plant Sci.* 179 364–373. 10.1016/j.plantsci.2010.06.006

[B22] Patterson H. D. Thompson R. (1971). Recovery of Inter-Block Information when Block Sizes are Unequal. *Biometrika* 58 545–554. 10.2307/2334389

[B23] Prapertchob P. Bhandari H. Pandey S. (2007). *Economic costs of drought and rice farmers’ drought-coping mechanisms in northeastern Thailand.* Los Banos: International Rice Research Institute (IRRI).

[B24] Rao A. N. Wani S. P. Ramesha M. S. Ladha J. K. (2017). *Rice Production Systems.* Cham: Springer.

[B25] Sandhu N. Kumar A. (2017). Bridging the Rice Yield Gaps under Drought: QTLs, Genes, and their Use in Breeding Programs. *Agronomy* 7:27. 10.3390/agronomy7020027

[B26] Seyoum M. Alamerew S. Bantte K. (2012). Genetic Variability, Heritability, Correlation Coefficient and Path Analysis for Yield and Yield Related Traits in Upland Rice (Oryza sativa L.). *J. Plant Sci.* 7 13–22. 10.3923/jps.2012.13.22

[B27] Solis J. Gutierrez A. Mangu V. Sanchez E. Bedre R. Linscombe S. (2018). Genetic Mapping of Quantitative Trait Loci for Grain Yield under Drought in Rice under Controlled Greenhouse Conditions. *Front. Chem.* 5:129. 10.3389/fchem.2017.00129 29359127 PMC5766644

[B28] Swamy B. P. M. Shamsudin N. A. A. Rahman S. N. A. Mauleon R. Ratnam W. Ma T. (2017). Association Mapping of Yield and Yield-related Traits Under Reproductive Stage Drought Stress in Rice (Oryza sativa L.). *Rice* 10:21. 10.1186/s12284-017-0161-6 28523639 PMC5436998

[B29] Venuprasad R. Lafitte H. R. Atlin G. N. (2007). Response to Direct Selection for Grain Yield under Drought Stress in Rice. *Crop Sci.* 47 285–293. 10.2135/cropsci2006.03.0181 34798789

[B30] Vikram P. Swamy B. M. Dixit S. Ahmed H. U. Teresa Sta, Cruz M. (2011). *qDTY 1.1*, a major QTL for rice grain yield under reproductive-stage drought stress with a consistent effect in multiple elite genetic backgrounds. *BMC Genet.* 12:89. 10.1186/1471-2156-12-89 22008150 PMC3234187

[B31] Wopereis M. C. S. Kropff M. J. Maligaya A. R. Tuong T. P. (1996). Drought-stress responses of two lowland rice cultivars to soil water status. *Field Crops Res.* 46 21–39. 10.1016/0378-4290(95)00084-4

[B32] Wu X. H. Wang W. Yin C. M. Hou H. J. Xie K. J. Xie X. L. (2017). Water consumption, grain yield, and water productivity in response to field water management in double rice systems in China. *PLoS One* 12:e0189280. 10.1371/journal.pone.0189280 29216292 PMC5720698

[B33] Xiong W. Holman I. Lin E. Conway D. Jiang J. Xu Y. (2010). Climate change, water availability and future cereal production in China. *Agricult. Ecosyst. Env.* 135 58–69. 10.1016/j.agee.2009.08.015

[B34] Yan T. Wang J. Huang J. (2015). Urbanization, agricultural water use, and regional and national crop production in China. *Ecol. Model.* 318 226–235. 10.1016/j.ecolmodel.2014.12.021

[B35] Zeigler R. S. Puckridge D. W. (1995). Improving sustainable productivity in rice-based rainfed lowland systems of South and Southeast Asia. *GeoJournal* 35 307–324. 10.1007/BF00989138

