# Supplementary material for: Capturing Genetic Variability and Identification of Promising Drought-Tolerant Lines in Exotic Landrace Derived Population Under Reproductive Drought Stress in Rice
Source: Front Plant Sci. 2022 Feb 14;13:814774. doi: 10.3389/fpls.2022.814774 (PMC8882627; doi:10.3389/fpls.2022.814774)
Supplement: Supplementary file 1 [file Table_1.docx]

**Supplementary Materials:**

**Table S1.** Correlation between yield and its related traits evaluated in Kasturi x Chao Khaw derived mapping population in two years (WS2015 and WS2016) non-stress as well as in drought-stress conditions.

| **Environment** | **Trait** | **DTF** | **PHT** | **NPT** | **PL** | **BM** | **HI** | **TGP** | **TFGP** | **SPF%** | **PY** |
| --- | --- | --- | --- | --- | --- | --- | --- | --- | --- | --- | --- |
| 2015_NSE | *DTF* | 1 |  |  |  |  |  |  |  |  |  |
| 2016_NSE | *DTF* | 1 |  |  |  |  |  |  |  |  |  |
| **2015_DSE** | *DTF* | 1 |  |  |  |  |  |  |  |  |  |
| **2016_DSE** | *DTF* | 1 |  |  |  |  |  |  |  |  |  |
| 2015_NSE | *PHT* | -0.15 | 1 |  |  |  |  |  |  |  |  |
| 2016_NSE | *PHT* | 0.18^*^ | 1 |  |  |  |  |  |  |  |  |
| **2015_DSE** | *PHT* | -0.29^**^ | 1 |  |  |  |  |  |  |  |  |
| **2016_DSE** | *PHT* | -0.39^**^ | 1 |  |  |  |  |  |  |  |  |
| 2015_NSE | *NPT* | 0.03 | 0.02 | 1 |  |  |  |  |  |  |  |
| 2016_NSE | *NPT* | -0.2 | -0.25^**^ | 1 |  |  |  |  |  |  |  |
| **2015_DSE** | *NPT* | 0.05 | -0.01 | 1 |  |  |  |  |  |  |  |
| **2016_DSE** | *NPT* | -0.12 | 0.02 | 1 |  |  |  |  |  |  |  |
| 2015_NSE | *PL* | 0 | 0.51^**^ | 0.11 | 1 |  |  |  |  |  |  |
| 2016_NSE | *PL* | 0.01 | 0.30^**^ | -0.07 | 1 |  |  |  |  |  |  |
| **2015_DSE** | *PL* | -0.24^**^ | 0.43^**^ | -0.05 | 1 |  |  |  |  |  |  |
| **2016_DSE** | *PL* | -0.26^**^ | 0.71^**^ | -0.02 | 1 |  |  |  |  |  |  |
| 2015_NSE | *BM* | 0.22^**^ | 0 | -0.02 | 0.1 | 1 |  |  |  |  |  |
| 2016_NSE | *BM* | 0.09 | 0.05 | -0.12 | -0.03 | 1 |  |  |  |  |  |
| **2015_DSE** | *BM* | 0.04 | -0.12 | 0.01 | -0.09 | 1 |  |  |  |  |  |
| **2016_DSE** | *BM* | 0.02 | -0.07 | -0.12 | -0.09 | 1 |  |  |  |  |  |
| 2015_NSE | *HI* | -0.21^**^ | 0.1 | 0 | -0.04 | -0.67^**^ | 1 |  |  |  |  |
| 2016_NSE | *HI* | -0.17^*^ | -0.02 | 0.15 | 0 | -0.41^**^ | 1 |  |  |  |  |
| **2015_DSE** | *HI* | -0.01 | 0.1 | 0 | 0.07 | -0.29^**^ | 1 |  |  |  |  |
| **2016_DSE** | *HI* | 0.03 | 0.03 | 0.14 | 0.07 | -0.73^**^ | 1 |  |  |  |  |
| 2015_NSE | *TGP* | 0.02 | 0.04 | -0.02 | 0.01 | 0.01 | 0.04 | 1 |  |  |  |
| 2016_NSE | *TGP* | 0 | -0.07 | -0.04 | 0.05 | -0.03 | 0.05 | 1 |  |  |  |
| **2015_DSE** | *TGP* | -0.09 | 0.06 | 0.01 | 0.01 | 0.13 | 0.01 | 1 |  |  |  |
| **2016_DSE** | *TGP* | -0.07 | 0.3 | 0 | 0.29 | -0.08 | 0.04 | 1 |  |  |  |
| 2015_NSE | *TFGP* | 0.04 | 0.06 | -0.04 | 0.01 | -0.01 | 0.07 | 0.93^**^ | 1 |  |  |
| 2016_NSE | *TFGP* | 0 | -0.06 | -0.01 | 0.02 | 0.01 | 0.01 | 0.89^**^ | 1 |  |  |
| **2015_DSE** | *TFGP* | -0.09 | 0.04 | 0.02 | -0.02 | 0.12 | -0.02 | 0.94^**^ | 1 |  |  |
| **2016_DSE** | *TFGP* | -0.1 | 0.41^**^ | 0.05 | 0.36^**^ | -0.07 | 0.06 | 0.82^**^ | 1 |  |  |
| 2015_NSE | *SPF%* | 0.02 | 0.09 | -0.06 | 0.01 | -0.04 | 0.08 | -0.03 | 0.34^**^ | 1 |  |
| 2016_NSE | *SPF%* | 0.01 | 0.00 | 0.05 | -0.08 | 0.09 | -0.09 | -0.14 | 0.31^**^ | 1 |  |
| **2015_DSE** | *SPF%* | -0.01 | -0.04 | 0.04 | -0.07 | -0.01 | -0.11 | -0.06 | 0.26^**^ | 1 |  |
| **2016_DSE** | *SPF%* | -0.04 | 0.25^**^ | 0.09 | 0.16^*^ | -0.01 | 0.06 | -0.11 | 0.48^**^ | 1 |  |
| 2015_NSE | *PY* | 0.02 | 0.08 | -0.05 | 0.1 | 0.43^**^ | 0.32^**^ | 0.08 | 0.08 | 0.02 | 1 |
| 2016_NSE | *PY* | 0 | 0.07 | 0 | -0.03 | 0.49^**^ | 0.55^**^ | 0.02 | 0.02 | -0.01 | 1 |
| **2015_DSE** | *PY* | 0.02 | -0.04 | -0.02 | -0.03 | 0.65^**^ | 0.51^**^ | 0.14 | 0.12 | -0.08 | 1 |
| **2016_DSE** | *PY* | 0.08 | 0.01 | 0 | -0.01 | 0.22^**^ | 0.42^**^ | 0.02 | 0.09 | 0.11 | 1 |

**DTF:** Days to flowering (days); **PHT:** Plant height (cm); **NPT:** Number of productive tillers; **PL:** Panicle length (cm); **PY:** Plot yield (g); **BM:** Biomass (g); **HI:** Harvest index (%); **TGP:** Total number of grains per panicle; **TFGP:** Total number of filled grains per panicle; **SPF%:** Spikelet fertility percentage. ‘*’and ‘**’ Significant at p < 0.05 and p < 0.01, respectively.

**Table S2.** Promising lines identified across the well-watered conditions of 2015 WS and 2016 WS having yield-advantage than mega variety IR 64

| **Sl. No.** | **Promising lines** | **DTF** | **PHT** | **NPT** | **PL** | **PY** | **BM** | **HI** | **TGP** | **TFGP** | **SPF%** | **GY (Kg/ha)** | |  |
| --- | --- | --- | --- | --- | --- | --- | --- | --- | --- | --- | --- | --- | --- | --- |
| 1 | IR 128807-82-B | 98 | 96.65 | 7 | 22.52 | 352.65 | 673.9 | 53.1 | 161 | 140 | 87.17 | | 4364 | |
| 2 | IR 128807-96-B | 95 | 95.19 | 6 | 22.65 | 345.1 | 675.9 | 51.84 | 175 | 154 | 88.71 | | 4289 | |
| 3 | IR 128807-11-B | 100 | 109.31 | 7 | 23.04 | 385.35 | 732.7 | 52.62 | 167 | 148 | 90.1 | | 4269 | |
| 4 | IR 128807-74-B | 95 | 104.28 | 8 | 23.45 | 336.15 | 631.6 | 54.22 | 164 | 141 | 85.76 | | 4219 | |
| 5 | IR 128807-175-B | 98 | 93.21 | 8 | 22.76 | 335.7 | 623.45 | 53.47 | 149 | 130 | 86.69 | | 4217 | |
| 6 | IR 128807-17-B | 95 | 101.94 | 8 | 22.67 | 332.4 | 594.9 | 54.57 | 142 | 126 | 87.71 | | 4200 | |
| 7 | IR 128807-14-B | 101 | 98.84 | 8 | 22.58 | 337.2 | 632.45 | 52.8 | 180 | 170 | 94.28 | | 4198 | |
| 8 | IR 128807-42-B | 99 | 98.79 | 7 | 23.42 | 380.35 | 689.8 | 54.14 | 151 | 134 | 88.45 | | 4165 | |
| 9 | IR 128807-22-B | 96 | 100.24 | 7 | 22.93 | 328.95 | 692.5 | 48.31 | 150 | 138 | 91.85 | | 4143 | |
| 10 | IR 128807-127-B | 92 | 96.08 | 8 | 22.69 | 329.25 | 662.4 | 50.51 | 184 | 151 | 83.43 | | 4143 | |
| 11 | IR 128807-100-B | 97 | 102.58 | 7 | 22.46 | 326.9 | 597.1 | 54.1 | 160 | 142 | 89.71 | | 4136 | |
| 12 | IR 128807-5-B | 99 | 101.01 | 7 | 22.44 | 373.25 | 661.85 | 55.72 | 181 | 151 | 84.29 | | 4113 | |
| 13 | IR 128807-99-B | 97 | 94.91 | 7 | 23.57 | 326.85 | 675.45 | 49.58 | 160 | 147 | 92.7 | | 4111 | |
| 14 | IR 128807-3-B | 99 | 94.51 | 7 | 22.67 | 330.45 | 1124.6 | 35.64 | 158 | 142 | 89.72 | | 4102 | |
| 15 | IR 128807-12-B | 97 | 101.95 | 8 | 22.8 | 324 | 637.5 | 50.85 | 175 | 138 | 79.04 | | 4082 | |
| 16 | IR 128807-46-B | 96 | 102.7 | 8 | 23.25 | 327.75 | 579.75 | 55.38 | 181 | 160 | 89.77 | | 4068 | |
| 17 | IR 128807-57-B | 99 | 102.57 | 8 | 23.58 | 334.75 | 679.55 | 49.98 | 182 | 163 | 90.29 | | 4062 | |
| 18 | IR 128807-58-B | 98 | 105.88 | 7 | 22.9 | 323.35 | 663.3 | 49.28 | 140 | 124 | 87.03 | | 4062 | |
| 19 | IR 128807-155-B | 96 | 102.72 | 8 | 23.48 | 321.4 | 582.75 | 54.25 | 139 | 121 | 85.24 | | 4061 | |
| 20 | IR 128807-75-B | 96 | 97.71 | 6 | 22.95 | 327.9 | 659.2 | 50.51 | 156 | 134 | 86.72 | | 4021 | |
| 21 | IR 128807-90-B | 96 | 99.67 | 8 | 23 | 318.85 | 579.3 | 54.24 | 169 | 145 | 86.32 | | 4011 | |
| 22 | IR 128807-147-B | 96 | 92.29 | 8 | 22.35 | 317.6 | 648.15 | 49.41 | 160 | 143 | 88.25 | | 3992 | |
| 23 | IR 128807-73-B | 99 | 98.79 | 8 | 23.57 | 316.6 | 630.5 | 50.37 | 202 | 166 | 85.23 | | 3968 | |
| 24 | IR 128807-129-B | 99 | 96.54 | 7 | 22.3 | 317.65 | 623.55 | 50.93 | 167 | 151 | 91.2 | | 3965 | |
| 25 | IR 128807-16-B | 97 | 102.31 | 8 | 23.35 | 327.65 | 665.35 | 51.05 | 146 | 126 | 85.1 | | 3962 | |
| 26 | IR 128807-38-B | 95 | 99.97 | 7 | 22.76 | 324.55 | 595.95 | 54.5 | 164 | 139 | 84.38 | | 3950 | |
| 27 | IR 128807-108-B | 98 | 103.66 | 7 | 23.96 | 322.75 | 653.45 | 50.19 | 149 | 129 | 86 | | 3950 | |
| 28 | IR 128807-116-B | 94 | 98.64 | 9 | 23.09 | 324.75 | 620.25 | 52.54 | 173 | 154 | 90.41 | | 3931 | |
| 29 | IR 128807-163-B | 96 | 99.51 | 7 | 22.67 | 348.85 | 621.7 | 54.34 | 141 | 117 | 80.75 | | 3892 | |
| 30 | IR 128807-103-B | 95 | 100.99 | 6 | 23.35 | 317.35 | 722.5 | 45.55 | 168 | 144 | 86 | | 3867 | |
| 31 | IR 128807-39-B | 95 | 96.93 | 8 | 23.44 | 316.95 | 601.45 | 53.17 | 163 | 138 | 84.6 | | 3866 | |
| 32 | IR 128807-121-B | 99 | 101.64 | 9 | 23.88 | 304.8 | 591.9 | 52.51 | 144 | 126 | 85.95 | | 3800 | |
| 33 | IR 128807-109-B | 95 | 95.45 | 7 | 22.55 | 341.65 | 599.1 | 55.48 | 154 | 129 | 82.9 | | 3778 | |
| 34 | IR 128807-95-B | 95 | 101.95 | 8 | 22.9 | 309.8 | 628.6 | 50.1 | 159 | 144 | 91.27 | | 3775 | |
|  | **Mean** | 97 | 99.69 | 7 | 23 | 332.04 | 654.48 | 51.62 | 162 | 141 | 87.26 | | 4051 | |
|  | **Min** | 92 | 92.29 | 6 | 22.3 | 304.8 | 579.3 | 35.64 | 139 | 117 | 79.04 | | 3775 | |
|  | **Max** | 101 | 109.31 | 9 | 23.96 | 385.35 | 1124.6 | 55.72 | 202 | 170 | 94.28 | | 4364 | |
|  | Chao Khaw (Donor parent) | 91 | 103.05 | 6 | 23.675 | 229.15 | 504.7 | 45.56 | 155.75 | 141.05 | 90.095 | | 2865 | |
|  | IR 64 (Check) | 92 | 79.035 | 9 | 21.72 | 288.25 | 594.1 | 49.34 | 171.65 | 157.1 | 91.265 | | 3603 | |
|  | Kasturi (Recipient parent) | 106 | 100.11 | 10 | 26.07 | 276.45 | 628.15 | 45.84 | 185.75 | 168.65 | 90.875 | | 3456 | |
|  | Shabhagidhan (Drought tolerant check) | 95 | 101.675 | 8 | 23.745 | 272.55 | 571.35 | 48.9 | 179.45 | 164.3 | 91.56 | | 3407 | |

**DTF:** Days to 50% flowering (days); **PHT:** Plant height (cm); **NPT:** Number of productive tillers; **PL:** Panicle length (cm); **PY:** Plot yield (g); **BM:** Biomass (g); **HI:** Harvest index (%); **TGP:** Total number of grains per panicle; **TFGP:** Total number of filled grains per panicle; **SPF%:** Spikelet fertility percentage; **GY:** Yield in Kg/h

**Table S3**. Promising lines identified for grain yield under reproductive stage drought stress condition of 2015 WS and 2016 WS having yield advantage over popular drought-tolerant check, Shabhagidhan and drought donor Chao Khaw

| **Sl. No.** | **Promising lines** | **DTF** | **PHT** | **NPT** | **PL** | **PY** | **BM** | **HI** | **TGP** | **TFGP** | **SPF%** | **GY (Kg/ha)** |
| --- | --- | --- | --- | --- | --- | --- | --- | --- | --- | --- | --- | --- |
| 1 | IR 128807-153-B | 90 | 78.24 | 6 | 20.88 | 154.8 | 477.1 | 34.33 | 122 | 66 | 54.47 | 1934.5 |
| 2 | IR 128807-126-B | 91 | 82.39 | 6 | 21.22 | 153 | 282.05 | 54.06 | 134 | 77 | 57 | 1912.5 |
| 3 | IR 128807-12-B | 90 | 79.55 | 6 | 21.17 | 149.5 | 304.05 | 49.15 | 121 | 69 | 56.61 | 1868.5 |
| 4 | IR 128807-145-B | 90 | 79.24 | 5 | 21.15 | 145.4 | 358.35 | 42.29 | 119 | 68 | 57.58 | 1818 |
| 5 | IR 128807-161-B | 89 | 80.41 | 6 | 21.92 | 145.25 | 305.95 | 48.14 | 126 | 69 | 55.44 | 1815.5 |
| 6 | IR 128807-114-B | 90 | 80.42 | 5 | 21.13 | 139.65 | 488.5 | 30.75 | 120 | 66 | 55.6 | 1745.5 |
| 7 | IR 128807-175-B | 87 | 80.98 | 6 | 21.75 | 139.45 | 284.35 | 48.31 | 122 | 70 | 56.8 | 1743 |
| 8 | IR 128807-138-B | 92 | 81.3 | 5 | 21.33 | 139.35 | 268.45 | 53.5 | 111 | 64 | 58.22 | 1741.5 |
| 9 | IR 128807-95-B | 90 | 80.14 | 7 | 21.56 | 139.3 | 286.4 | 48.7 | 122 | 71 | 58.31 | 1741.5 |
| 10 | IR 128807-54-B | 93 | 77.05 | 5 | 21.54 | 138.95 | 283.5 | 50.12 | 117 | 61 | 53.09 | 1737 |
| 11 | IR 128807-11-B | 96 | 76.02 | 5 | 20.77 | 138.7 | 273.2 | 51.02 | 124 | 65 | 52.51 | 1733 |
| 12 | IR 128807-134-B | 87 | 84.34 | 6 | 21.64 | 138.4 | 265.85 | 53.01 | 129 | 69 | 53.24 | 1730 |
| 13 | IR 128807-163-B | 90 | 81.24 | 6 | 21.23 | 136.85 | 430.05 | 34.07 | 122 | 66 | 54.13 | 1711 |
| 14 | IR 128807-97-B | 89 | 79 | 5 | 21.32 | 136.45 | 490.2 | 30.59 | 127 | 68 | 53.15 | 1705.5 |
| 15 | IR 128807-164-B | 90 | 77.95 | 6 | 21.19 | 136.3 | 329.6 | 43.27 | 120 | 66 | 55.12 | 1704 |
| 16 | IR 128807-39-B | 89 | 78.75 | 6 | 21.42 | 135.9 | 277.65 | 49.25 | 137 | 80 | 57.75 | 1698.5 |
| 17 | IR 128807-35-B | 87 | 79.27 | 6 | 21.2 | 135.7 | 292.05 | 47.57 | 151 | 84 | 55.02 | 1697 |
| 18 | IR 128807-112-B | 88 | 84.98 | 6 | 21.66 | 134.4 | 252.5 | 52.36 | 136 | 77 | 56.03 | 1680 |
| 19 | IR 128807-139-B | 87 | 82.55 | 6 | 21.62 | 133.65 | 428.3 | 37.34 | 126 | 70 | 55.45 | 1671 |
| 20 | IR 128807-2-B | 93 | 77.47 | 7 | 21.1 | 133.15 | 255.7 | 51.17 | 114 | 63 | 55.93 | 1664.5 |
| 21 | IR 128807-106-B | 86 | 80.81 | 6 | 20.86 | 132.95 | 364.75 | 37.71 | 131 | 71 | 54.07 | 1662 |
| 22 | IR 128807-152-B | 93 | 80.9 | 6 | 21.59 | 132.8 | 310.65 | 42.77 | 127 | 62 | 49.82 | 1659.5 |
| 23 | IR 128807-40-B | 92 | 82.38 | 5 | 21.19 | 132.3 | 290.2 | 46.8 | 124 | 69 | 54.93 | 1654 |
| 24 | IR 128807-49-B | 89 | 81.65 | 5 | 21.57 | 131.9 | 264.4 | 50.38 | 122 | 69 | 56.04 | 1649 |
| 25 | IR 128807-174-B | 89 | 83.92 | 5 | 21.61 | 131.8 | 272.8 | 48.9 | 127 | 74 | 57.76 | 1647.5 |
| 26 | IR 128807-132-B | 94 | 73.2 | 6 | 20.38 | 130.25 | 467.2 | 29.76 | 119 | 70 | 58.73 | 1628 |
| 27 | IR 128807-111-B | 92 | 74.68 | 5 | 21.17 | 130.2 | 265.45 | 49.85 | 123 | 62 | 51.04 | 1627.5 |
| 28 | IR 128807-18-B | 93 | 78.85 | 6 | 20.61 | 130.1 | 251.6 | 52.62 | 114 | 63 | 55.69 | 1626.5 |
| 29 | IR 128807-107-B | 90 | 79.15 | 6 | 21.32 | 130.05 | 265.25 | 50.57 | 131 | 74 | 55.78 | 1625 |
| 30 | IR 128807-16-B | 89 | 85.2 | 6 | 21.58 | 128.75 | 265.95 | 49.66 | 131 | 74 | 56.53 | 1609.5 |
| 31 | IR 128807-36-B | 90 | 82.34 | 6 | 21.04 | 128.75 | 437.35 | 32.16 | 117 | 68 | 57.41 | 1609.5 |
| 32 | IR 128807-27-B | 88 | 89.96 | 5 | 21.98 | 128.1 | 301.75 | 44.47 | 133 | 76 | 56.66 | 1601 |
| 33 | IR 128807-99-B | 88 | 85.7 | 7 | 21.01 | 127.95 | 255.3 | 52.03 | 125 | 71 | 56.53 | 1600 |
| 34 | IR 128807-80-B | 92 | 87.25 | 5 | 21.34 | 127.65 | 265.4 | 48.59 | 125 | 68 | 53.97 | 1595.5 |
| 35 | IR 128807-34-B | 90 | 83.84 | 5 | 22.1 | 126.45 | 388.95 | 34.07 | 130 | 72 | 54.9 | 1580.5 |
| 36 | IR 128807-55-B | 89 | 78.13 | 6 | 21.55 | 125.45 | 293.55 | 44.78 | 123 | 68 | 55.31 | 1568.5 |
| 37 | IR 128807-45-B | 89 | 79.49 | 5 | 21.16 | 125.2 | 415.1 | 31.34 | 115 | 62 | 54.3 | 1565 |
| 38 | IR 128807-76-B | 87 | 85.21 | 5 | 21.9 | 124.85 | 239.75 | 52.6 | 141 | 80 | 56.17 | 1561 |
| 39 | IR 128807-110-B | 91 | 82.65 | 6 | 21.11 | 124.35 | 272.8 | 45.9 | 127 | 72 | 56.13 | 1554.5 |
| 40 | IR 128807-166-B | 88 | 82.52 | 7 | 20.73 | 124.25 | 233.7 | 53.86 | 127 | 69 | 54.05 | 1553.5 |
| 41 | IR 128807-70-B | 89 | 91.56 | 6 | 22.21 | 123.65 | 369.25 | 34.56 | 120 | 69 | 57.75 | 1545.5 |
| 42 | IR 128807-38-B | 91 | 83.31 | 6 | 22.28 | 123 | 259.25 | 49.09 | 132 | 73 | 55.37 | 1538 |
| 43 | IR 128807-60-B | 89 | 76.96 | 6 | 22.87 | 122.9 | 297.7 | 45.25 | 121 | 64 | 53.73 | 1536.5 |
| 44 | IR 128807-44-B | 90 | 79.72 | 8 | 21.32 | 118.55 | 334.75 | 36.53 | 117 | 64 | 54.61 | 1482 |
| 45 | IR 128807-155-B | 90 | 80.09 | 6 | 20.91 | 118.4 | 284.5 | 41.95 | 103 | 57 | 55.81 | 1480 |
| 46 | IR 128807-24-B | 91 | 79.45 | 5 | 20.69 | 118.05 | 286.2 | 41.67 | 116 | 64 | 55.79 | 1476 |
| 47 | IR 128807-5-B | 96 | 79.25 | 5 | 20.47 | 117.95 | 274.05 | 43.63 | 120 | 68 | 57.07 | 1474.5 |
| 48 | IR 128807-46-B | 94 | 85.18 | 6 | 22.26 | 115.65 | 249.75 | 47.42 | 122 | 67 | 54.99 | 1446 |
|  | **Mean** | 90 | 81.14 | 6 | 21.36 | 132.01 | 314.82 | 44.75 | 124 | 69 | 55.46 | 1650 |
|  | **Min** | 86 | 73.2 | 5 | 20.38 | 115.65 | 233.7 | 29.76 | 103 | 57 | 49.82 | 1446 |
|  | **Max** | 96 | 91.56 | 8 | 22.87 | 154.8 | 490.2 | 54.06 | 151 | 84 | 58.73 | 1935 |
|  | Chao Khaw (Donor parent) | 84.95 | 67.375 | 5.8355 | 21.275 | 96.85 | 200.9 | 48.76 | 140.75 | 79.465 | 56.1 | 1210 |
|  | IR 64 (Check) | 89.23 | 68.485 | 5.065 | 20.655 | 49.3 | 145.35 | 34.575 | 138.1 | 61.145 | 46.475 | 616.5 |
|  | Kasturi (Recipient parent) | 98.44 | 85.53 | 5.3705 | 22.37 | 51.25 | 157.65 | 34.05 | 140.55 | 57.87 | 41.44 | 640.5 |
|  | Shabhagidhan (Drought tolerant check) | 90.045 | 65.46 | 6.4515 | 22.34 | 75.2 | 194.45 | 38.965 | 137.7 | 71.095 | 51.47 | 940 |

**DTF:** Days to 50% flowering (days); **PHT:** Plant height (cm); **NPT:** Number of productive tillers; **PL:** Panicle length (cm); **PY:** Plot yield (g); **BM:** Biomass (g); **HI:** Harvest index (%); **TGP:** Total number of grains per panicle; **TFGP:** Total number of filled grains per panicle; **SPF%:** Spikelet fertility percentage; **GY:** Yield in Kg/ha
